# Supplementary figures and images for: External factors influence intrinsic differences in Stx2e production by Porcine Shiga Toxin-producing Escherichia coli strains
Source: PLoS Pathog. 2025 Oct 22;21(10):e1013616. doi: 10.1371/journal.ppat.1013616 (PMC12571312; doi:10.1371/journal.ppat.1013616)

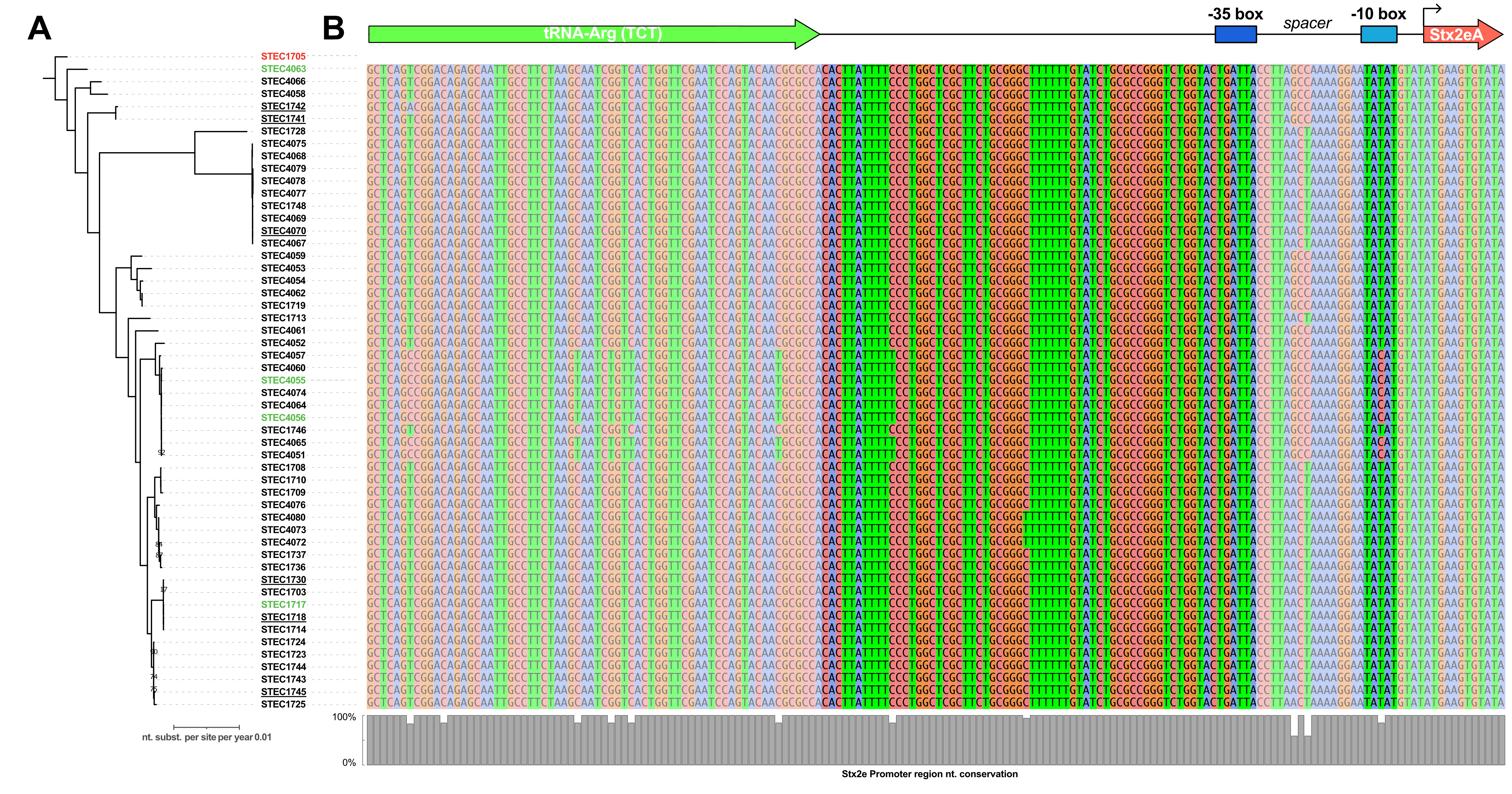

Supplement: S1 Fig — (A) Maximum-Likelihood (ML) phylogenetic tree of all included STEC strains (n = 52), including strain 1705 as negative control and outgroup (red). Bootstraps <95 are indicated next to branches. Underlined strains were used in an in-depth characterization of the impact of external factors and strains highlighted in green were used in holin deletion experiments. (B) Polymorphisms identified in the downstream promoter region as predicted by sigma70pred “scan” (0.92 SVC score), highlighting the -35 box, spacer, -10 TATA box, and Stx2eA codon start. (TIFF) [file ppat.1013616.s001.tiff]
