# Supplementary material for: External factors influence intrinsic differences in Stx2e production by Porcine Shiga Toxin-producing Escherichia coli strains
Source: PLoS Pathog. 2025 Oct 22;21(10):e1013616. doi: 10.1371/journal.ppat.1013616 (PMC12571312; doi:10.1371/journal.ppat.1013616)
Supplement: Attachment — Submitted filename: Response to the reviewers.docx [file ppat.1013616.s011.docx]

**Response to the Reviewers' Comments:**

Reviewer's Responses to Questions

**Part I - Summary**
Please use this section to discuss strengths/weaknesses of study, novelty/significance, general execution and scholarship.

**Reviewer #1:** Production and secretion of shiga-like toxin stx2b in 57 porcine STEC isolates were studied. The main conclusions are that variations in secretion of toxin in response to antibiotic and environmental stressors are in some isolates dependent on presence of a holin gene downstream of the stxAB genes. Deletion of the holin gene convincingly showed that production/secretion of stx2b was reduced and in complemented isolates the production/secretion was increased.
The authors also showed that deoxycholic acid affected different isolates in different ways, either by up or downregulation of secretion while catecholamines had no effect. Addition of submic antibiotics ciprofloxacine and enrofloxacine also induced stx secretion in a subset of isolates. The tested substances were chosen due to reported ability to induce the SOS response.
The paper is nicely written and very interesting, but I do have some concerns and questions.

**Reviewer #2:** This study addresses an important topic related to Shiga toxin-producing E. coli (STEC) in pigs, focusing on the factors influencing stx2e toxin production and secretion. The work combines phenotypic assays with genomic data, which is a strength. The topic is highly relevant for both understanding STEC pathogenesis and informing potential therapeutic strategies.

However, the study currently has several weaknesses that limit its impact. The manuscript does not fully utilize the available long-read sequencing data, restricting the genomic analysis to 10 kb flanking regions without deeper exploration of prophage structures, plasmid associations, or structural variation. In addition, there are issues with clarity and consistency throughout the manuscript, particularly in the description of methods, results, and figure presentations.

Despite these issues, the study has the potential to make a meaningful contribution to the field if the authors address the concerns raised, clarify and expand their genomic analyses, and improve the organization and clarity of the manuscript.

Reply: We would like to take the opportunity to thank the reviewers for their time and efforts in reviewing our manuscript. Their comments have further strengthened our manuscript.

**Part II – Major Issues: Key Experiments Required for Acceptance**
Please use this section to detail the key new experiments or modifications of existing experiments that should be absolutely required to validate study conclusions.

Generally, there should be no more than 3 such required experiments or major modifications for a "Major Revision" recommendation. If more than 3 experiments are necessary to validate the study conclusions, then you are encouraged to recommend "Reject".

**Reviewer #1:** The authors conclude that secretion of stx2b is highly variable but almost all produced stx2b is also secreted (fig 1 and suppl table 7), hence the results are not linked to secretion but to production and thereby probably regulation of expression.

Reply: We agree with the reviewer that Stx2e production varies between strains and that this is most likely caused by a differential regulation of toxin expression. Nevertheless, Stx2e secretion levels vary between strains and as such the conclusion that secretion varies still holds true. To avoid confusion between the process of secretion or the transport of the toxin through the inner and outer membrane and the presence of Stx2e in the extracellular environment, we have changed 'secretion' to 'secretion levels' where applicable.

In Fig 5A the growth at sub MIC levels of the antibiotics are analyzed and all are significantly different i.e downregulated compared to the control as indicated with three stars, this is not apparent from the data in the figure, is it correct and if so, a downregulation in growth might affect other factors than what the authors intended to analyze.
Required experiment: Could the authors complement the studies with analysis of toxin production over time and growth phase with and without sub-MIC antibiotics.

Reply: Yes, this is correct.

We wanted to investigate how sub-MIC levels of the tested antibiotics affected Stx2e secretion levels. We expected some effect on bacterial growth, even at 1/4th of the MIC of the tested antibiotics and based on the mode of action of the tested antibiotics we also expected a decreased Stx2e secretion for some of the antibiotics. Amoxicillin for instance inhibits the biosynthesis and repair of the bacterial cell wall and while growth was inhibited, Stx2e secretion levels were unaffected. In contrast, tetracycline and erythromycin, which inhibit bacterial protein synthesis, inhibited bacterial growth (but less so than the other antibiotics) and as expected also decreased Stx2e secretion levels. The fluoroquinolones ciprofloxacin and enrofloxacin inhibit bacterial DNA replication and are known to trigger the bacterial SOS response. In other STEC strains this has been shown to result in inducing the lytic phase of prophages and subsequently to increased release of Shiga toxin. Our genome analysis however revealed that none of the strains had intact prophages. Remarkably, toxin secretion was increased in a subset of strains when cultured in the presence of fluoroquinolones at 1/4th of the MIC, even when growth was reduced.

The reviewer proposed to perform an additional experiment to analyze Stx2e production and bacterial growth over time. While this experiment makes sense, we faced methodological and strain-specific hurdles preventing us from performing this experiment. An earlier study showed that the production of Stx2 subtypes is delayed as compared to Stx1 (de Mena et al., 1997, Rev Argent Microbiol) and that production of Stx2 subtypes is at its maximum during the stationary phase. A preliminary analysis revealed that the tested strains reached the stationary phase after 6h and that we could not detect Stx2e secretion during the log phase of bacterial growth by ELISA. Based on these preliminary results, we decided not to perform the proposed kinetic study.

Also in fig 5B cipro and enro gives two different subpopulations, how was the statistical analysis performed to take this into account since apparently one subpopulation has no change in response to these two antibiotics. In this figure the same 17 isolates that were upregulated by cipro apparently had a significant downregulation in response to tetra and ery but where they analysed separately from the other isolates and did the others not change? Please clarify.

Reply: Thank you for pointing this out. We have extended our description of the data analysis. To analyse the effect of the tested antibiotics on Stx2e release we performed a paired non-parametric Friedman test to check for significant differences between the control and test condition for all strains. We did not analyze strains separately.

The authors continue to analyze the 17 isolates that had increased production/secretion in response to cipro. A holin gene was found downstream in all isolates but that was also found in totally 38 isolates, please clarify the names of the 17 isolates and indicate them in fig 2. Also please clarify if there is any correlation between the response to cipro and deoxycholate upregulation/downregulation in these 17 isolates as well as in all isolates.

Reply: Figure 2 has been revised to include the strain IDs. To avoid further complexity, we did not indicate the 17 strains that respond to the fluoroquinolones with increased Stx2e release. We provide that information in Table S1. This table gives an overview of all data, including the response of the strains to the presence of bile acids and fluoroquinolones in terms of Stx2e secretion levels. The response to deoxycholate was tested in 6 strains (3 that showed increased and 3 that showed decreased Stx2e secretion levels in response to bile acids) and therefore we did not evaluate a potential correlation between the strains that responded to deoxycholate and those that responded to the fluoroquinolones. As shown in table S1, there is no correlation between the strains that responded to fluoroquinolones and those that responded to bile acids.

The holin mutant and complemented mutant experiments are convincing but since secretion and expression are so similar in these isolates it might be involved in transcriptional or translational regulation rather than secretion.
required analysis: The study should be complemented with an analysis of the promoter regions of stxAB for all isolates to determine if there are differences in binding sites for transcription factors that correlate with responses to deoxyxholate and cipro. In addition, gene expression analysis of stx2b in presence and absence of holin can be analysed to determine if holin affects gene expression of stx2b

Reply: We have analyzed the promotor region of the Stx2e operon in more depth for all strains (supplementary figure S1 and see below). We extracted the upstream region of the *stx2eA* coding sequence and aligned this region for subsequent analysis. To predict the upstream promoter in this region, we subjected the sequences to the Sigma70Pred tool (<https://webs.iiitd.edu.in/raghava/sigma70pred/>), which predicted the highlighted promoter region with a high Support Vector Classifier (SVC) of 0.92 as compared to other “random” sequences (ranging between 0 and 0.08 SVC). Only three polymorphic positions were identified in the promoter region, including (i) T>C within the -10 TATA box, (ii) T>C within the spacer region, and (iii) A>G within the spacer region. These mutations are closely linked to the phylogenetic relatedness of our tested STEC strains and do not show any correlation with the observed Shiga toxin secretion levels.

Since Stx2e production and secretion levels are very well correlated in these strains, we are confident that fig. 6B shows that the presence of holin does not affect Stx2e secretion levels in the absence of enrofloxacin. As such, holin does not influence Stx2e transcription-translation in these strains. On the contrary, our results are in line with the known function of holins in the bacteriophage lytic cycle, that is to make pores in the inner membrane to facilitate the transport of endolysins, proteins that degrade the cell wall.

Figure S1.

**Reviewer #2:** Expanded genomic analysis using long-read data: The current analysis is limited to 10 kb flanking regions around the stx2e genes. A more comprehensive characterization using the long-read assemblies is necessary. Specifically, the authors should clarify whether stx2e is plasmid- or chromosome-encoded, perform detailed prophage characterization, and explore structural variations or broader synteny surrounding the stx2e operon.

Reply: We agree that it might seem as if the long-read data was not used to the fullest in this manuscript. Though, to maintain focus, we have only included the 10 kb flanking regions of the *Stx2e* gene as more extended analyses did not show any associations or links with high or low secretors. As indicated in the Materials & Methods and available in Supplementary files S4-S6, we have performed a whole genome association study to identify potential genetic targets associated with the observed phenotypes. This did not result in any clear associations. Also, as mentioned in the introduction (L61-L63, L147), genes encoding Shiga toxins, including Stx2e, are present in the bacterial chromosome. Likewise, none of the identified *Stx2e* genes were located on plasmids as has been described in our previous manuscript (10.3389/fmicb.2023.1139312). We made sure to clarify the chromosomal origin of the *Stx2e* genes throughout the revised manuscript. In addition, as mentioned in the M&M (L271-L273), Phaster was used to identify and characterize prophages. While prophage genes were found scattered throughout the bacterial chromosome, intact prophages were absent. We have included a brief description in the Results section (L399-401).

We do agree that performing more in-depth structural analyses or synteny surrounding the *Stx2e* gene operon would be interesting, though this requires fully circularized genomes for each of the genomes at study. This is not available for all isolates within the current manuscript. These isolates were sequenced in the context of a different project to study the diagnostic potential of long-read sequencing for ETEC/STEC strains (10.3389/fmicb.2023.1139312). As a result, not all genomes had even coverage (a typical observation when using R9.4.1 flow cells and the SQK-RBK009 library preparation), which resulted in some genomes with a higher number of contigs, hence not being fully circularized (See supplementary Table S3). Also, the existence of long repeats and/or complex regions (*e.g.,* composite transposons), could hamper a full resolution of bacterial WGS data even with high(er) coverage long-read sequencing. As such, we maintained focus throughout our manuscript by representing the genetic landscape within 10 kb up- and downstream of the *Stx2e* operon.

Clarification and complete description of genomic methods: The Materials and Methods section should include a clear description of the genome assembly, quality control procedures, SNP calling, and phylogenetic tree construction. The authors should specify the software versions, settings used, and explain whether the phylogenetic tree was based on SNPs from the core genome and how SNPs were defined.

Reply: thank you for this suggestion. We gave a full description in our previous manuscript. To avoid copying exact methodologies of our previously performed analyses, we have included the following sentence on the genomic analyses (L252-L253):

“Isolation of DNA, library preparation, sequencing, and genome assembly were performed as described [35].”

Reference 35 refers to our previous manuscript (10.3389/fmicb.2023.1139312) in which all genomes were assembled. The same genome assemblies were used in the current manuscript, without any modifications. This reference includes all details on how gDNA was obtained, sequencing libraries were prepared, and how genomes were assembled, including genome QC. Please see the detailed information below. Due to the extensive detailed explanation (including references and software versions) in our previous manuscript, we believe repeating these methodologies is redundant.

For your reference, below you can find an excerpt from the Materials and Methods of our previous manuscript:

2.3. Long-read whole-genome nanopore sequencing

For long-read nanopore sequencing, an aliquot of each culture was thawed and inoculated in 10 mL LB medium for overnight incubation. The next day, 4 mL was centrifuged at 16,000 × *g* to collect all bacterial cells. Pellets were resuspended in 250 μL dPBS prior to the isolation of high molecular weight (HMW) DNA using the ZymoBIOMICS DNA MiniPrep kit (Zymo Research) at the PathoSense laboratory. Manufacturer’s instructions were followed, with the addition of a 30-min Proteinase K treatment (20 μg.μL^−1^; Promega) after cellular disruption using a TissueLyser (Qiagen) for twice 5 min at 30 oscillations per minute. Quality of the HMW DNA was verified using a NanoDrop Spectrophotometer. If A_260_/A_230_ or A_260_/A_280_ were below 1.7, DNA was subjected to an additional clean-up using CleanNGS (CleanNA) magnetic beads in a 1:1 ratio. High quality HMW DNA was subjected to sequencing using 400 ng DNA input per isolate in a rapid library preparation (SQK-RBK004; ONT). A maximum of 10 isolates were multiplexed on a single R.9.4.1 flow cell. Sequencing was done on a GridION device (ONT), allowing raw data demultiplexing and collection in the MinKNOW software (ONT). To evaluate nanopore sequencing quality and accuracy, DNA from an *E. coli* ATCC 25922 strain was isolated and sequenced in the same workflow as described before. This was done in triplicate to address final genome completeness and accuracy as determined against the *E. coli* K ATCC 25922 genome (NZ_CP009072.1) using CheckM (based on 1,212 genes from 27 *E. coli* reference genomes) and pomoxis (ONT), respectively ([Vereecke et al., 2020](https://www.frontiersin.org/journals/microbiology/articles/10.3389/fmicb.2023.1139312/full#ref103), [2023](https://www.frontiersin.org/journals/microbiology/articles/10.3389/fmicb.2023.1139312/full#ref104); [Bokma et al., 2021a](https://www.frontiersin.org/journals/microbiology/articles/10.3389/fmicb.2023.1139312/full#ref10)). An overview of sequencing output and coverage can be found in [Supplementary Table S1](https://www.frontiersin.org/journals/microbiology/articles/10.3389/fmicb.2023.1139312/full#SM1).

2.4. Generation of complete circular and accurate ETEC/STEC genome assemblies

Resulting demultiplexed fast5 files were subjected to Bonito base calling v0.4.0 (ONT R&D base caller), using its *default* model (dna_r9.4.1@v3.3 model) on the Ghent University HPC Tier 2 GPU cluster Joltik using 1x GPU. The fastq files per isolate were used in an *in-house* bacterial whole-genome assembly pipeline using the Trycycler pipeline v0.5.3 ([Wick et al., 2021a](https://www.frontiersin.org/journals/microbiology/articles/10.3389/fmicb.2023.1139312/full#ref110)). In short, reads were filtered using filtlong v0.2.1 (--min_length 1,000 --keep_percent 95)[^1^](https://www.frontiersin.org/journals/microbiology/articles/10.3389/fmicb.2023.1139312/full#fn0006) prior to subsampling into 10 subsamples [--min_read_depth 50 --count 10 --genome_size 5 M ([Wick et al., 2021a](https://www.frontiersin.org/journals/microbiology/articles/10.3389/fmicb.2023.1139312/full#ref110))]. Each subsampled fastq file was used to perform an independent initial genome assembly using either flye v2.9 ([Kolmogorov et al., 2019](https://www.frontiersin.org/journals/microbiology/articles/10.3389/fmicb.2023.1139312/full#ref55)), raven v1.8.1 ([Vaser and Šikić, 2021](https://www.frontiersin.org/journals/microbiology/articles/10.3389/fmicb.2023.1139312/full#ref101)), miniasm_and_minipolish.sh v0.3,[^2^](https://www.frontiersin.org/journals/microbiology/articles/10.3389/fmicb.2023.1139312/full#fn0007) or wtdbg2 v1.12 ([Ruan and Li, 2020](https://www.frontiersin.org/journals/microbiology/articles/10.3389/fmicb.2023.1139312/full#ref88)) as instructed on the Trycycler manual page. Final assemblies were used as input for Trycycler to generate consensus genomes using *default* settings. This includes the removal of contigs with coverage below 10% of the median sample sequencing coverage, clustering, reconciling, multiple sequence alignment, read partitioning, and the generation of complete circular genome and plasmid consensuses. The latter were subjected to final read mapping and polishing using minimap2 v2.20 ([Li, 2018](https://www.frontiersin.org/journals/microbiology/articles/10.3389/fmicb.2023.1139312/full#ref61)) and medaka v1.5.0 (ONT), respectively. If no consensus assembly could be obtained from Trycycler (e.g., too contiguous assemblies), flye assemblies were used for mapping and polishing. These strains were indicated with an F at the end of the strain name. Contigs were classified to chromosomes and plasmids using ViralVerify v1.1.[^3^](https://www.frontiersin.org/journals/microbiology/articles/10.3389/fmicb.2023.1139312/full#fn0008) Completeness and accuracy of final consensus genomes was assessed using ribosomal multi-locus sequence typing (rMLST) ([Jolley et al., 2018](https://www.frontiersin.org/journals/microbiology/articles/10.3389/fmicb.2023.1139312/full#ref49)) and CheckM v1.1.0 ([Parks et al., 2015](https://www.frontiersin.org/journals/microbiology/articles/10.3389/fmicb.2023.1139312/full#ref77)). A genome was considered complete based on 1,212 marker genes from 27 *Escherichia* genomes. When all marker genes were identified, a completeness of 100% was reported. Genome QC reports and associated NCBI accession numbers can be found in [Supplementary Table S1](https://www.frontiersin.org/journals/microbiology/articles/10.3389/fmicb.2023.1139312/full#SM1).

Improved data presentation and figure clarity: Figures (particularly Figures 1 and 2) need revision to clearly show strain IDs, secretion levels, and genomic organizations. Categorization of strains into secretion levels (low, moderate, high) should be indicated consistently. Legends should be updated to match the figure contents, and visual clarity should be improved, for example by using standard tree layouts and adjusting color usage in the clinker plot.

Reply: Figure 1 has been revised and now includes strain IDs. The categorization of the strains into the defined Stx2e secretion levels is given in Table S1. Since this is of less importance for the other data sets and to avoid complexity, we did not indicate this categorization in figure 1 and 2.

Figure 2 was updated to include the secretion levels as an extra bar chart between the phylogenetic tree and the genetic landscape as well as an updated rectangular view with updated bootstrap placements. As requested, we have updated the color-space within the clinker plot in Fig. 2C by graying-out any major flanking regions and emphasizing CDS of interest in differential colors. We have updated the legend and made sure to clarify the identified mutations within the *Stx2e* genes as well in an extra panel in Fig. 2D (see below).

**Part III – Minor Issues: Editorial and Data Presentation Modifications**
Please use this section for editorial suggestions as well as relatively minor modifications of existing data that would enhance clarity.

**Reviewer #1:**

Line 103: remove ref 22

Reply: Reference 22 has been removed.

Line 109: ref 43? Should it be renumbered to 27? Or is one reference missing? Ref 43 is a python tool used to determine flanking regions. Check numbering of all references after addressing ref 43.

Reply: Thank you for pointing this out. We have corrected this mistake and added the correct reference.

Fig 5B, there are no bars in the figure.

Reply: Thank you for pointing this out. We have corrected this error.

**Reviewer #2:**

• References are not listed in order throughout the manuscript.

Reply: Thank you for pointing this out. We have corrected this.

• Some references are missing and should be added where relevant.

Reply: We have revised the reference list and corrected errors.

• Line 415 refers to the wrong table; it should refer to Table S7.

Reply: The reference to table S1 was correct. Table S7 gives the Stx2e production and secretion data (in ng/mL) of three independent experiments in the absence of additional stimuli. Based on other comments, we have revised table S1.

• Table S2 is not referred to in the text; please update.

Reply: We now refer to table S2 in the text (L205).

• The GWAS section should be removed, as it is not properly integrated or supported.

Reply: Considering the (major) comment on the use of our long-read datasets, we believe this part is of importance to note that no significant findings were observed when performing a GWAS. This allowed for an initial check for potential genomic content/contexts being associated with the observed phenotypes. Since long-read datasets were used, positional information of genes is available, the GWAS output could redirect us to (broader) genomic changes if identified and found significant. As such, this is considered an important step in how our experimental flow and interpretation was done.

• Was AMR (antimicrobial resistance) genomically analyzed in addition to experimental testing? Please clarify. Line 408 indicates this but no analysis is shown.

Reply: The focus of the current manuscript was the Stx2e production and secretion and not the AMR phenotypes, since this has been extensively studied in our previous manuscript (10.3389/fmicb.2023.1139312). The strains used in the current manuscript represent a subset of the strains that were presented in the previous manuscript as referenced above and discussed in the Materials & methods section.

• Analysis of fimbriae/adhesion molecules: many strains are negative for adhesion molecules in Table S1. Were factors beyond F4 and F18 checked?

Reply: Yes, the presence of additional virulence factors was evaluated. We kindly refer the reviewer to our previous manuscript (10.3389/fmicb.2023.1139312), which has been referenced in the current manuscript.

• Why focus only on F4 and F18 CFs in Table 1? Could other adhesion molecules be relevant?

Reply: We focus on F4 and F18 fimbriae as these fimbriae are most prevalent in strains causing porcine post-weaning diarrhea (F4+ or F18+) and edema disease (F18+).

• The number of strains varies between different analyses (58, 57, 53, 52). Please clarify these differences where relevant.

Reply: The differences were partially clarified in the Materials and Methods. We provided additional clarifications in the figure legends where appropriate. Figure 1 shows Stx2e secretion for 58 strains. One strain did not have the Stx2e operon and was used as a negative control (see table S1). Figures 3, 4 and 5A,B show the response of the STEC strains to bile acids, stress hormones and antibiotics. The control strain was not included in these analyses, resulting in 57 tested STEC strains. For the STEC genome analysis, 5 strains were excluded as mentioned in the Materials and Methods (L255-L256). Figure 2 then shows the genomic regions around the Stx2e operon of the remaining 53 strains, containing 52 STEC strains and one control strain (strain 1705).

• Clarify what is meant by "genomic regions" — does it refer to upstream, downstream, or all flanking regions shown in Figure 2?

Reply: It refers to sequences up- and downstream of the Stx2e operon as shown in figure 2. We further clarify this in the M&M (L262-L263).

• Checking for QseEF: no information is provided on methodology.

Reply: This information is provided in the Materials & Methods under the “STEC genome analysis” section. This includes the following information:

“Finally, the presence of genes known to be involved in bacterial responses to bile acids

and stress hormones was evaluated using abricate v1.0.1 [49] and run with --mincov --minid both set to 80 and 60 (Supplementary Table S8-S9).”

Table S8 contains the target genes that were screened for, while Table S9 contains the results of the genetic screening.

• Table S1: Include the year of isolation. Indicate whether isolates came from one farm or several farms per country.

Reply: All strains were isolated in 2022. Strains originating from one country were collected on several farms. We have clarified this in the legend of table S1.

• Table S3: Please add a strain ID column for easier cross-referencing.

Reply: The strain ID was given as 1-000xxxx. We have removed the '1-000' as suggested for easier cross referencing.

• Table S4 (GWAS output): Define Group 1 and Group 2 properly.

Reply: As requested we have now defined group 1 and 2 in the legend of the table and we also included a more detailed description of this in the Materials and methods (L266-271).

“For this analysis two different groups were created, based on low (<1 ng.mL^-1^; 11 strains), moderate (1-10 ng.mL^-1^; 41), or high (>10 ng.mL^-1^, 5) Stx2e production. A first analysis, group 1, included both high and moderate Stx2e producers as positive (*i.e.,* 1 in the GWAS metadata file in Supplemental Table S4), whereas the second analysis, group 2, only included the high Stx2e producers as positives (Supplementary Table S4)."

• Table S8: Include a description of contents.

Reply: As requested, we have now added a legend to clarify the content of Table S8. We have also revised this table to increase clarity.

• Table S9: Clarify the coverage and identity thresholds used in abricate analysis; the text states 80% coverage and 60% identity, but Table S9 lists 80% for both.

Reply: The Materials and methods sections states that both metrics (--mincov and --minid) were run both at 80% or 60%. This complies with a more strict and loose gene search to reassure identification of all genetic targets. Hence, two tables (80% ID, 80% Coverage and 60% ID, 60% Coverage) are presented in Supplementary Table S9. This has been further clarified in the text and the legend of the supplementary tables.

• Materials and Methods: Specify the number of biological and technical replicates, e.g., for ELISA experiments.

Reply: The biological replicates are specified in the figure legends. A biological replicate is defined as an independent culture of the bacteria on a separate day (Fig. 1, 3, 4, 5A, 5B, 6) or an individual animal (Fig. 5C). For the ELISAs, all samples were tested in duplicate as mentioned on L241.

• How were the groupings for level of secretion defined — based on cytotoxicity or previous publications? Clarify.

Reply: The groups were defined based on the Stx2e secretion levels as measured by ELISA. As mentioned in the results (L380-L382), strains could be divided in low, moderate and high Stx2e secreting strains. To perform the GWAS, strains were divided in low (0) and moderate/high Stx2e secreting strains (1) (group 1, Table S4). Since this analysis did not produce results, we also reassigned the strains in a second GWAS. In this second analysis (group 2, Table S4), low/moderate Stx2e secreting strains were assigned a zero, while high secreting strains were labeled as 1. We have clarified this in the legend of Table S4 in the revised manuscript. Also this second analysis did not produce meaningful results.

• Why only investigate stx2e? Clarify the scientific motivation.

Reply: We focused on Stx2e as we were interested in Shiga toxin-producing *E. coli* strains that cause disease in carrier animals. Most animals are asymptomatic carriers, however, in pigs, STEC strains cause edema disease. Around 99% of STEC strains associated with edema disease in pigs have Stx2e as the sole Shiga toxin (Baranzoni et al, 2016, Front Microbiol; Remfry et al., 2021, J Food Protection).

• Figure 1: The number of strains does not match the legend. Strain IDs are missing.

Consider making the bar horizontal and indicating strain IDs if possible.

Reply: The number of strains mentioned in the legend (58) does match the number of strains shown in the figure. The control strain (1705) did not produce or secrete Stx2e, so no data can be shown. We have added strain IDs to the figure and revised the figure legend.

• Figure 2:
A more standard phylogenetic tree layout (rectangular or slanted branches) would improve clarity, especially for readers comparing branch lengths and tip relationships.
Bootstrap values below 95% are currently plotted along the branches; place them next to internal nodes to avoid confusion and update the figure legend accordingly.
The clinker figure uses many colors which are not described; this makes interpretation difficult. Many genes of interest and flanking CDSs downstream of holin are colored purple, making them hard to distinguish. Remove unnecessary colors.
Add a scale ruler on top of the clinker figure to indicate the region length instead of using a scale bar.
Use colors to highlight stx2e genes carrying non-synonymous mutations instead of writing mutations as text, which is difficult to see.
Describe E46* clearly in the figure legend.
Highlight which strains are low, moderate, and high secretors in the clinker figure so that secretion levels can be cross-referenced with Figure 1.
In the updated Figure 2 with a bar graph, color the bars based on secretion level (low, mid, high).

It is mentioned in Figure 6 that different holin gene conformations exist, but this is not visible in Figure 2. Clarify differences between strains, e.g., strain 4055 and 4056.

Reply: Figure 2 was updated to include the secretion levels as an extra bar chart between the phylogenetic tree and genetic landscape as well as an updated rectangular view with updated bootstrap placements. Also, we have updated the color-space within the clinker plot in Fig. 2C by graying-out any major flanking regions and emphasizing CDS of interest in differential colors. We have updated the legend and made sure to clarify the identified mutations within the *Stx2e* genes as well in an extra panel in Fig. 2D.

Color coding the bars to indicate strains with a low, moderate or high secretion levels makes this figure too complicated.

The reviewer noticed that the holin gene configurations as shown in figure 6 are not visible in figure 2. This might be due to the use of different softwares to analyze the Stx2e operon and its surrounding region. The analysis in figure 2 represents an annotation of this region with Prokka, while for the holin gene configurations this region was annotated and analyzed using the PGAP annotation provided on NCBI from our submitted genomes. The PGAP annotation applies a more stringent annotation algorithm and certain coding sequences might be skipped or might be presented differently. We noticed the Materials & Methods did not mention this and has now been revised to clarify this (L279-281).

• Figure 3A–C: Clarify whether squares and circles represent different conditions.

Reply: The squares and circles do not represent different conditions but were added as an extra visual aid to indicate increased (circles) or decreased (squares) Stx2e secretion levels upon stimulation with bile acids.

• In bile component secretion assays (13 strains), either adjust the figure to include strain IDs or refer clearly to a supplementary table that lists concentration and outcome.

Reply: We have revised table S1 and added the Stx2e secretion levels in response to bile acids. We refer to this revised table S1 where appropriate.

• Figure 4: Recommend moving to Supplementary Figures for better flow. Also, the figure legend does not describe Figure 4B (fold change).

Reply: The figure legend has been revised to better describe the presented figures. Concerning the recommendation to move figure 4 to the supplementary data, we prefer to keep it here. In our opinion, this is relevant information and presenting it in the main text does not have a major impact on its readability. However, if the reviewer insists, we will follow this recommendation.

• Figure 5B: Bars are missing; please clarify or correct.

Reply: The figure legend has been revised.

• Line 371: The text states "three non-silent SNPs," but five mutations (D175E, T207A, P289L, T296K, S313P) are listed. Clarify and correct either the number of SNPs or the description of the mutations.

Reply: Thank you for pointing out this error. We have correct the number of SNPs.

• Recommend including a multiple sequence alignment (MSA) of the stx2e sequences as a supplementary figure to clearly show the non-synonymous SNPs.

Reply: This information has been included in Figure 2 as part of the extra clarification of the observed mutations within the *Stx2e* genes.

• Specify how many strains showed increased versus decreased stx2e secretion when tested with different bile components.

Reply: This information is mentioned in the text (L455-L457) for bile acids from bovine source. We also mentioned that we observed a similar response to bile acids from porcine source. The same strains showed an upregulated or downregulated Stx2e secretion or did not respond to porcine bile acids. We then selected three strains that showed increased or decreased Stx2e secretion levels to assess the impact of animal age on the response of the strain to bile acids (fig. 3C1 and C2). The same strains were then used to assess the response of the strains to individual bile acids (fig. 3 D1 and D2). We have clarified this further in the text (L473-L475).

• In the genomic context section, specify which genomic context is referred to and clarify if clusters are based on phylogenetic clades.

Reply: We have carefully read the text but could not identify the sentence(s) the reviewer is referring to. The clustering of the STEC strains in genomic contexts around the Stx2e operon is based on the build-up of their up- and downstream (*i.e.,* 10 kbp each side) CDS landscape. This information has been included in the legend of Figure 2 for clarification.

• Clarify how genomic regions were defined (e.g., 10,000 bp each side or total 10,000 bp?).

Reply: The genomic region was defined as 10,000 bp on each side of the *Stx2e* operon. We've clarified this in the M&M (L262-L263).

• Did you also look at larger genomic contexts beyond 10,000 bp? If so, please discuss findings.

Reply: We would like to refer the reviewer to our reply on the major comments.

• In the abstract, “potential new therapeutics” are mentioned — please elaborate briefly in the Discussion on what kinds of therapeutics the authors envision.

Reply: We added the potential new therapeutics to incentivize readers to think about this. We believe that elaborating on these new therapeutics in the discussion would guide the readers too much into certain directions but we also agree that without providing further details about these potential new therapeutics this sentence is somewhat irrelevant. As such, we deleted this part of the sentence in the abstract.

• Recommend shortening the Discussion and removing or minimizing references to GWAS results unless strongly supported.

Reply: We have critically reread our discussion. In our opinion, shortening the discussion would compromise the quality of our manuscript. We did took some efforts to write it more concisely. We only briefly touch upon the GWAS results (L608-612), or better the absence of GWAS results, and discuss what would be needed to potentially obtain results (L613-617).
