# Supplementary material for: External factors influence intrinsic differences in Stx2e production by Porcine Shiga Toxin-producing Escherichia coli strains
Source: PLoS Pathog. 2025 Oct 22;21(10):e1013616. doi: 10.1371/journal.ppat.1013616 (PMC12571312; doi:10.1371/journal.ppat.1013616)
Supplement: Attachment — Submitted filename: rebuttal letter round 2.docx [file ppat.1013616.s012.docx]

**Part I - Summary**
Please use this section to discuss strengths/weaknesses of study, novelty/significance, general execution and scholarship.

Reviewer #1: The ms has improved and is still very interesting in my opinion but there are still some items that needs correction or clarification.

Reply: we would like to thank the reviewer for his appreciation of our manuscript. We have carefully read the comments and addressed these below. We believe that by addressing these comments our manuscript has improved.

**Part II – Major Issues: Key Experiments Required for Acceptance**
Please use this section to detail the key new experiments or modifications of existing experiments that should be absolutely required to validate study conclusions.

Generally, there should be no more than 3 such required experiments or major modifications for a "Major Revision" recommendation. If more than 3 experiments are necessary to validate the study conclusions, then you are encouraged to recommend "Reject".

Reviewer #1: The levels of production/expression and secretion are nearly identical in fig1, hence all expressed stx2b are also secreted. But the authors argue that secretion itself is mediated by holin causing more holes or pores in the outer membrane thereby increasing secretion out of the bacterium. But check for example 1717 in presence of Enro, the induction of secreted toxin is increased from 7 to 116 ng and since all expressed/assembled toxin is also secreted through the outer membrane in normal conditions (as proved in fig 1) holin plus enro somehow induce increased levels of mature stx2bAB holotoxin, but only in presence of enro (by SOS responses). Since holin is located in the inner membrane and I assume stx2b is assembled in the periplasm maybe holin helps the A and B subunit to reach the periplasm. I suggest another term, why not use “stx2b assembly” or “production of mature holotoxin” I think using secretion and even secretion levels are not backed up by your results because more toxin is produced (and hence released).

Reply: we would like to emphasize that our manuscript concerns Stx2e and not Stx2b. We do not argue that holins create pores in the outer membrane. We clearly mentioned in the Results section (L567) and discussion (L689) that holins form pores in the inner membrane. We hypothesize that these holin pores might aid in Stx2e secretion by facilitating transport of the subunits across the inner membrane. We have added this latter part to the text (L569).

Hence, we fully agree with the reviewer's assumption that holin may aid Stx2e A and B subunits to reach the periplasm. While we appreciate the suggestions made by the reviewer, we would argue against the use of the proposed terms in the results section as holins do not directly assist in assembly (it has no chaperone function) nor does it increase production. However, we now understand the concerns of the reviewer related to the use of 'secretion' and 'secretion levels'. Since we measured Stx2e levels in the bacterial culture supernatant upon adding external factors to the strains, we would like to keep the focus on what we've measured. As such, we have replaced 'secretion' and 'secretion levels' with 'production', 'presence in the extracellular environment' or 'increased extracellular Stx2e levels' throughout the manuscript.

In the discussion, we speculate on the involved mechanisms (L700-714). Since we showed that adding fluoroquinolones to the bacteria results in an increased presence of Stx2e in the extracellular environment and that this only happens when a holin gene is present directly downstream of the Stx2e operon in the correct orientation, we rephrased the part on the holins as follows: Holin might enable increased transport of Stx2e A and/or B subunits across the inner membrane, leading to an increased presence of Stx2e in the extracellular environment. We also included the suggestion of the reviewer on holotoxin assembly and further indicate that currently it is unresolved how Stx2e is transported across the outer membrane.

Figure 2 has improved substantial with the changes but mark the 17 isolates that show the enro response, I cannot see the reason not to and it helps the reader.
There is a discrepancy in strains used that needs to be addressed Fig S1 and fig 2 mark holin analysed strains in green, i.e 4056, 4064 and 4057 and 1717, these are also mentioned in the text line 338 (4056, 4064, 4057 Δholin, and 1717 Δholin)
Then compare line 563-564: To test this, the lambda red recombination system was used to replace the holin gene with a kanamycin resistance cassette (KanR) in two strains (4056 and 1717).
Line 568-571: Additionally, we transformed these deletion mutants and two strains (4055 and 4063) that were previously not inducible by enrofloxacin with a plasmid containing the holin gene under the control of an inducible promotor. Hence different isolates are mentioned
4056 is responsive to enro according to table S1 but appears genetically identical to 4064 and 4055 who are unresponsive but do have a holin gene at the same place. Why would complementation of holin work for 4064 and 4055? And what isolates were used? only 1717 is consistent.

Reply: we now indicate the 17 strains in fig. 2. We thank the reviewer for the attention to detail and for pinpointing the discrepancies in strain IDs in the figures, tables and text. We couldn't not immediately identify the origin of these discrepancies in the text and data files and thus, we checked our -80°C stock for the strains that were modified. We confirmed that strains 1717 and 4056 were used to delete the holin gene, while strains 4055 and 4063 were used to supplement the holin gene (as indicated in figure 6). We have corrected the strain IDs in the text and in figure 2. Apparently typing errors in the first drafts of the manuscript resulted in a mismatch of strain IDs, which were taken along in the next versions. We apologise for not spotting these earlier. Figure 6a and the text at (L561-564; L589) provide an explanation why holin supplementation in these strains resulted in increased extracellular Stx2e levels.

The new analysis of the promoter region is nice but only include -35 and -10 regions, transcription factors probably bind upstream of -35 so can the region be expanded 5´

Reply: we have extended the sequence analysis to identify potential differences in upstream transcription factor binding sites. However, as shown in the figure below, we could not identify differences, except for strain 1746. In our opinion, this difference in strain 1746 does not explain the observed variation in extracellular Stx2e levels. We have added this to the manuscript as Figure S1. We extended our analysis even further upstream of the Stx2e operon and found differences in two subsets of strains. However, also these differences do not explain the observed variation in extracellular Stx2e levels between the strains.

**Part III – Minor Issues: Editorial and Data Presentation Modifications**
Please use this section for editorial suggestions as well as relatively minor modifications of existing data that would enhance clarity.

Reviewer #1: I disagree with the discussion about PCR assays for holin presence since it does not say anything, holin is there also in unresponsive isolates. If there is a difference in sequences or holin promoter region this must be addressed and explained in eg fig2

Reply: In the context of the paper we found it evident that we meant sequencing and not simple PCR assays to check for the presence of a holin gene. We realize that it was not obvious and rephrased this part again to clarify this point (L720-L725). We indicate differences in fig. 6a, showing that unresponsive isolates do not have the holin gene in the correct orientation or not immediately downstream of the Stx2e operon. We cannot show these differences in figure 2 as they only became apparent when analysing genome sequences with NCBI gene viewer (Fig. 6) and not with Clinker (Fig. 2), as mentioned in our previous rebuttal letter in response to a comment from the other reviewer. For you convenience, we've added this comment and our response below.

*Comment of Reviewer 2:*

*It is mentioned in Figure 6 that different holin gene conformations exist, but this is not visible in Figure 2.*

*reply: The reviewer noticed that the holin gene configurations as shown in figure 6 are not visible in figure 2. This might be due to the use of different softwares to analyze the Stx2e operon and its surrounding region. The analysis in figure 2 represents an annotation of this region with Prokka, while for the holin gene configurations this region was annotated and analyzed using the PGAP annotation provided on NCBI from our submitted genomes. The PGAP annotation applies a more stringent annotation algorithm and certain coding sequences might be skipped or might be presented differently. We noticed the Materials & Methods did not mention this and has now been revised to clarify this.*
